# Severe Bilateral Breast Mucinous Carcinoma with Bilateral Lungs and Cutaneous Metastasis: A Case Report and Literature Review

**DOI:** 10.1155/2018/8759564

**Published:** 2018-03-06

**Authors:** Rong Pu, Yanchu Li, Xianyong Li

**Affiliations:** ^1^Oncology Department, Chengdu Fuxing Hospital, Chengdu, China; ^2^Oncology Department, West China Hospital of Sichuan University, Chengdu, China

## Abstract

The case of a female who had severe, rare, terminal breast mucinous carcinoma (BMC) and failed to receive surgery and chemotherapy was reported. The patient was diagnosed with pure BMC (ER++, PR++, CerbB-2−, and Ki-67 10%) accompanied with bilateral lungs, bilateral chest walls with skin ulcer (*D* = 14 cm), lymph nodes of bilateral armpits, and right supraclavicular metastases. ECOG (Eastern Cooperative Oncology Group) and NRS (Numeric Rating Scale) pain scores were 4 and 6, respectively. Because the patient refused traditional chemotherapy and radiotherapy on religious grounds, an herbal medicine containing *Panax ginseng*, *Agrimonia pilosa*, and white flower *Patrinia* herb was administered; extensive nursing for tumor debridement was also provided. Quality of Life (QOL) improved and pain reduced. Tumor-bearing survival time was prolonged. The present case dictates that herbal extract medicines and supportive treatment can be helpful for uncommon severe BMC as an appropriate alternative treatment.

## 1. Introduction

Breast mucinous carcinoma (BMC) is a rare histologic type breast cancer [1, 2]. This cancer, except for the micropapillary pattern, usually occurs in elderly patients and presents a relatively good prognosis compared with more common types of breast cancer [3]. Soo Youn Bae's study reported that the 5-year disease-free survival (DFS) and overall survival (OS) rates for MC were 95.2% and 98.9%, respectively [4]. However, details of its natural history are not clear. Treatment guidelines for BMC are mostly extrapolated from data on the basis of infiltrating ductal carcinoma (IDC) [5, 6]. Thus, standard breast cancer operations and chemotherapy are recommended [7, 8]. However, once the patient refuses surgery and all chemotherapy, the options are limited, as in this case.

Recently, no large series of palliative care therapies for mucinous carcinoma has been reported. Here, we report the case of a woman who had terminal stage BMC with bilateral lungs and chest wall metastases and received herbal extract medicines.

## 2. Case Presentation

**Figure 1:**
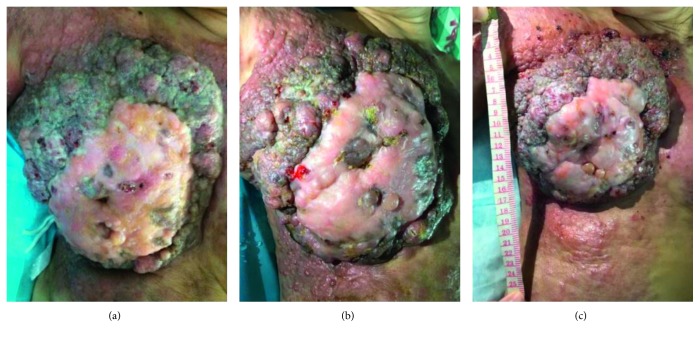
Breast and chest wall invasion accompanied by ulcer and infection are shown. (a) BMC after one week of treatment, on September 30, 2016; (b) BMC after one month of treatment, on October 18, 2016; (c) BMC after four months of treatment, on January 11, 2017.

**Figure 2:**
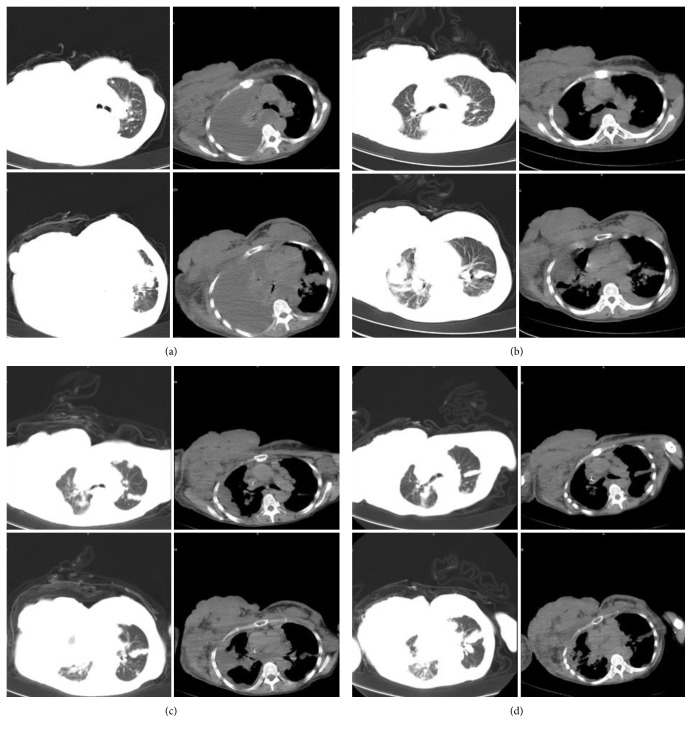
CT scan showed bilateral lung metastasis and pleural fluid. (a) Massive right pleural fluid and bilateral breast metastasis were detected before treatment on September 22, 2016; (b) massive right pleural fluid mostly disappeared, and bilateral breast metastasis was stable after three weeks of treatment, on October 14, 2016; (c) No further observation of right pleural fluid was made, and bilateral breast metastasis was stable after four months of treatment, on January 26, 2017; (d) No further occurrence of right pleural fluid or lung metastasis was noted, and bilateral breast metastasis was stable after seven months of treatment, on April 27, 2017.

A 51-year-old woman was diagnosed with BMC at the Sichuan Cancer Hospital & Institute (Chengdu, China) on June 2015, and the pathological results showed ER++ 90%, PR++ 90%, CerbB-2−, CK5/6−, EGFR−, and Ki-67 10%. Positron emission tomography combined with computed tomography (PET/CT) scan showed metastasis on the bilateral lungs, lymph nodes of bilateral armpits, and right supraclavicular lymph nodes. Because she was a Buddhist, the patient refused to receive surgery, chemotherapy, and endocrine therapy; her past medical history was significant only for nursing and acupuncture treatment. On the contrary, abnormal acupuncture accelerated cancer progression gradually and externally. Eight months after diagnosis, the biggest tumor measured 15.0 × 19.0 × 16.0 cm, and the portion ulcerating through the skin measured 14.0 × 6.0 × 8.0 cm. The large tumor had already occupied the bilateral breasts, and the bilateral chest walls and abdominal wall were invaded (Figure 1); computed tomography (CT) showed bilateral pleural fluid, multilung metastasis, right pulmonary vein invasion, and pericardium invasion (Figure 2). Meanwhile, the patient experienced dyspnea and cachexia, and ECOG (Eastern Cooperative Oncology Group) and NRS (Numeric Rating Scale) pain scores were 4 and 6, respectively. Hemoglobin concentration had decreased to 55.0 g/L which was attributed to continuous bleeding from the tumor's surface. The serum carbohydrate antigen CA15-3 concentration was more than 1000 U/mL (0–25 U/mL), and carcinoembryonic antigen (CEA) concentration was 7.77 ng/mL (0–5.093 ng/mL). The patient was expected to survive for less than one month.

This patient was treated four times daily by an oral herbal extract medicine, which contains *Panax ginseng*, *Agrimonia pilosa*, and white flower *Patrinia* herb, and comprehensive debridement nursing once a day since September 22, 2016. The herbal extract preparation has been approved by the China Food and Drug Association (CFDA).

**Figure 3:**
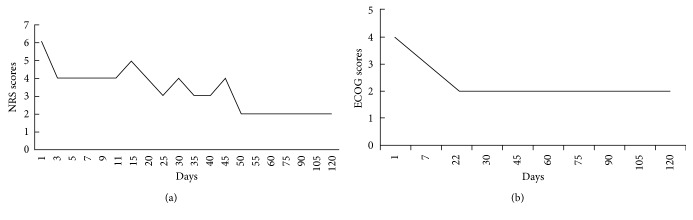
(a) NRS score showed that the pain had improved gradually and dramatically after 11 days of treatment. (b) ECOG score showed that QOL had improved and remained stable.

After four months of treatment, serum CA15-3 concentration fell to within normal limits and remained at that level. QOL has significantly improved, and tumor-bearing living has been achieved for 10 months from September 22, 2016. Bilateral breast and chest invasion was stable (Figures 1 and 1). Massive pleural fluid almost disappeared (Figures 2–2) from October 14, 2016, to April 27, 2017, and the patient has almost recovered from dyspnea and cachexia. Meanwhile, ECOG and NRS pain scores decreased to 2 and 2 (Figure 3), respectively. CT scan showed that the primary tumor was stable, and no new metastasis occurred (Figures 2–2) from October 14, 2016, to April 27, 2017. Meanwhile, hematological side effects greater than grade 2 according to Common Terminology Criteria for Adverse Events 3.0 (CTCAE 3.0) were not observed; however, mild constipation related to herbal drugs and skin erythema caused by hyperthermia were observed.

Herbal extract medicines and supportive care improved the patient's QOL, eased severe symptoms, and prolonged survival time.

## 3. Discussion

Mucinous carcinoma is relatively rare and tends to occur in the middle-aged or elderly (average 62.6 years, range 8–87 years) [9, 10] and is generally considered to have a good prognosis [11]. The proportion of mucinous carcinoma among all primary breast cancers has been reported to be 1% to 4%. In previous studies, locally advanced and distant metastasis cases were rare [12]. Rasmussen reported 247 cases of BMC that varied in size from 0.5 cm to 12 cm, with an average diameter of 2.8 cm, and only 11 patients (4.5%) experienced skin invasion [13]. However, Ishikawa et al. reported an extremely large tumor of about 25 cm in diameter [14]; a similar case was reported by Murakami et al. [15], and the patient had an 8-year survival due to systemic chemotherapy, intra-arterial infusion chemotherapy, and radiotherapy.

The favorable prognosis of BMC is usually based on surgery and chemotherapy, even radiation therapy. However, sometimes, patients are already in stage IV at diagnosis; they may have cachexia, or the first or second round of treatments based on surgery, chemotherapy, and radiotherapy may have failed. Such patients cannot tolerate toxicity. Sometimes, just as in this case, patients refuse chemotherapy and surgery. The anticancer ability of herbal medicine has been recently proved, especially as palliative care. Thus, for these patients, herbal extract medicines, which provide acceptable effects and low toxicity, could be a priority choice.

Our herbal extract medicines are extracted from *Panax ginseng*, *Agrimonia pilosa*, and white flower *Patrinia* herb, which contains polysaccharides, and have been used on late-stage breast cancer, lung cancer, pancreatic cancer, and liver cancer [16–18]. Meanwhile, depending on our fundamental research, 3-(4,5-dimethylthiazol-2-yl)-2,5-diphenyltetrazolium bromide (MTT) and crystal violet assay indicated that the ingredients of herbal extract medicines can inhibit 4T1 breast cancer and CT26 colon cell line growth (unpublished data).

Above all, the result indicated that specific and individual palliative care and herbal medicines had a good improvement and outcome for terminal BMC, and it could be an alternative method with low toxicity. The significance of this case lies in prolonging the tumor-bearing survival and improving QOL with herbal extract medicines for those patients who fail to receive or tolerate common treatment. Because side effects are limited and toxicity is low, terminal BMC could be viewed as a chronic disease, obviating an unnecessary intervention with high cell toxicity.

## 4. Conclusion

Besides traditional chemotherapy and surgery, individualized treatments for terminal stage BMC patients are necessary, and selecting appropriate treatments based on patients' body condition is crucial. Herbal medicines with limited toxicity may be the recommended initial treatment for patients with terminal cancer.

## References

[1] Dhillon R., Depree P., Metcalf C., Wylie E. (2006). Screen-detected mucinous breast carcinoma: potential for delayed diagnosis. *Clinical Radiology*.

[2] Ohashi R., Sakatani T., Matsubara M. (2016). Mucinous carcinoma of the breast: a comparative study on cytohistological findings associated with neuroendocrine differentiation. *Cytopathology*.

[3] Barbashina V., Corben A. D., Akram M., Vallejo C., Tan L. K. (2013). Mucinous micropapillary carcinoma of the breast: an aggressive counterpart to conventional pure mucinous tumors. *Human Pathology*.

[4] Bae S. Y., Choi M.-Y., Cho D. H., Lee J. E., Nam S. J., Yang J.-H. (2011). Mucinous carcinoma of the breast in comparison with invasive ductal carcinoma: clinicopathologic characteristics and prognosis. *Journal of Breast Cancer*.

[5] Di Saverio S., Gutierrez J., Avisar E. (2008). A retrospective review with long term follow up of 11,400 cases of pure mucinous breast carcinoma. *Breast Cancer Research and Treatment*.

[6] Lacroix-Triki M., Suarez P. H., MacKay A. (2010). Mucinous carcinoma of the breast is genomically distinct from invasive ductal carcinomas of no special type. *Journal of Pathology*.

[7] Dumitru A., Procop A., Iliesiu A. (2015). Mucinous breast cancer: a review study of 5 year experience from a hospital-based series of cases. *A Journal of Clinical Medicine*.

[8] Thurman S. A., Connolly J. L., Schnitt S. J. (2003). Outcome after breast-conserving therapy for patients with stage I or II musinous, medullary, or tubular breast carcinoma. *International Journal of Radiation Oncology Biology Physics*.

[9] Cao A. Y., He M., Liu Z. B. (2012). Outcome of pure mucinous breast carcinoma compared to infiltrating ductal carcinoma: a population-based study from China. *Annals of Surgical Oncology*.

[10] Chen H. S., Chen F. M., Yang S. F., Hsu J. S. (2014). Primary cutaneous mucinous carcinoma of the breast. *Kaohsiung Journal of Medical Sciences*.

[11] Yoneyama F., Tsuchie K., Sakaguchi K. (2003). Massive mucinous carcinoma of the breast untreated for 6 years. *International Journal of Clinical Oncology*.

[12] Zhang L., Jia N., Han L., Yang L., Xu W., Chen W. (2015). Comparative analysis of imaging and pathology features of mucinous carcinoma of the breast. *Clinical Breast Cancer*.

[13] Rasmussen B. B. (1985). Human mucinous breast carcinomas and their lymph node metastases. A histological review of 247 cases. *Pathology-Research and Practice*.

[14] Ishikawa T., Ichikawa Y., Shimura M. (2002). Locally advanced mucinous carcinoma of the breast with sudden growth acceleration: a case report. *Japanese Journal of Clinical Oncology*.

[15] Murakami M., Sano A., Okamoto Y., Nishikawa T., Nishimura S., Matsusue S. (2001). Validity of local treatment including intraarterial infusion chemotherapy and radiotherapy for fungating adenocarcinoma of the breast: case report of more than 8-year survival. *American Journal of Clinical Oncology*.

[16] Pu R., Zhao Q., Li Z. (2016). Rapid bone repair in a patient with lung cancer metastases to the spine using a novel herbal medicine: a case report. *Oncology Letters*.

[17] Li Y., Li X., Tip P., Zhang L. (2015). Use of a novel herbal medicine in a 75-year-old woman with multi-metastatic pancreatic cancer: a case report and review of the literature. *Oncology Letters*.

[18] Li Xianyong L. Y., Weihua Y., Lingyan Z. (2008). Application of Bionic internal control treatment for malignant cancer. *Oncology Progress*.

